# Extended Reality Gaming for Exercise and Mindfulness Throughout Pediatric Cancer Rehabilitation: Protocol for a Randomized Controlled Trial

**DOI:** 10.2196/64879

**Published:** 2024-12-23

**Authors:** Byron Lai, Kelli Chaviano, Joshua S Richman, Mahmoud Ahmad, Ashley Wright, Raven Young, Drew Davis, James H Rimmer, Avi Madan-Swain, Joseph H Chewning

**Affiliations:** 1 Division of Pediatric Rehabilitation Medicine Department of Pediatrics University of Alabama at Birmingham Birmingham, AL United States; 2 Department of Surgery University of Alabama at Birmingham Birmingham, AL United States; 3 Dean's Office School of Health Professions University of Alabama at Birmingham Birmingham United States; 4 Division of Pediatric Hematology-Oncology Department of Pediatrics University of Alabama at Birmingham Birmingham, AL United States

**Keywords:** oncology, rehabilitation, bone marrow transplant, virtual reality, VR, physical activity, exercise, extended reality, XR, pediatric cancer

## Abstract

**Background:**

Pediatric patients with cancer have limited options to self-manage their health while they are undergoing treatments in the hospital and after they are discharged to their homes. Extended reality (ER) using head-mounted displays has emerged as an immersive method of improving pain and mental health and promoting health-enhancing physical activity among a variety of clinical groups, but there is currently no established protocol for improving both physical and mental health in pediatric cancer rehabilitation.

**Objective:**

This phase I, pilot, feasibility randomized controlled trial aims to investigate the potential effects of a 14-week ER program on physical activity participation and indicators of health among pediatric patients with cancer who undergo bone marrow transplantation. An ancillary aim is to evaluate the feasibility of the program through participant engagement.

**Methods:**

This study includes a 2-arm parallel group design with a 1-group crossover (the control group will start the intervention after a waiting period). Overall, 16 pediatric patients with cancer undergoing rehabilitation (aged ≥8 years) at a children’s hospital will be randomly allocated into one of two groups: (1) an immediate start group that undergoes an ER program in the hospital until discharge and then for 8 weeks at home (total duration of approximately 14 weeks), and (2) a waitlist control group that undergoes usual care in the hospital and for 8 weeks at home, before receiving the 8-week home ER program. The program will include active video gaming with rhythmic music exercises as well as mindfulness-based practices using a high-quality app. Home-based programming will include behavioral coaching calls. Physical activity will be measured daily through step counts using a tri-axial accelerometer. Health outcomes will be measured across time and include global health, measured by the National Institutes of Health Patient-Reported Outcomes Measurement Information System (PROMIS) Pediatric Global Health Scale Short Form 7+2, and lung function, measured by a forced expiratory volume using a peak flow meter. Feasibility will be evaluated through participant engagement metrics, such as enrollment, dropout, adverse events, and attendance rates. Descriptive statistics will be obtained for all study variables. Outcomes will be modeled using mixed modeling procedures, and changes in means will be estimated with CIs.

**Results:**

The study was funded in February 2024. Recruitment procedures started on June 27, 2024. All data are anticipated to be collected by February 2026. Full trial results are anticipated to be analyzed and submitted for publication by March 2026. The study’s anticipated end date is March 31, 2026.

**Conclusions:**

This trial tests an accessible remote program for improving both physical and mental health among pediatric patients with cancer. The knowledge obtained from this study will inform the development of a larger trial.

**Trial Registration:**

ClinicalTrials.gov NCT06298357; https://clinicaltrials.gov/study/NCT06298357

**International Registered Report Identifier (IRRID):**

DERR1-10.2196/64879

## Introduction

### Overview of Pediatric Cancer and Physical Activity

Pediatric cancer is estimated to impact roughly 400,000 children and adolescents aged 0 to 19 years worldwide annually [1]. In the United States, it is estimated that there are about 77,000 new diagnoses each year, making up approximately 5% of all cancer cases [2]. Pediatric cancer encompasses malignancies occurring in individuals aged 19 years and younger, affecting various organs or tissues, which necessitate specialized diagnosis, treatment, and long-term follow-up care [3-5]. Notably, pediatric cancer and its treatments can diminish physical activity in children, resulting in reduced mobility, muscle weakness, fatigue, and impaired physical function [6]. Reduced activity increases their vulnerability to accelerated frailty, secondary diseases, mortality, and mental health issues, all of which can be mitigated through regular physical activity participation [7-9]. Disappointingly, physical activity levels among pediatric cancer survivors are far lower than those observed among the general pediatric population [10], which places them at high risk for cardiometabolic disease [11,12].

### Role of Aerobic Exercise in Bone Marrow Transplant Rehabilitation

Bone marrow transplant (BMT) is an effective option for treatment among patients with hematological disorders, including leukemia [13,14]. However, this treatment has been associated with poor outcomes, specifically decreased cardiorespiratory capacity for months after treatment [15,16], and this leads to compromised rehabilitation, participation in daily activities, and quality of life. A systematic review has found 25 randomized controlled trials (RCTs) demonstrating that exercise is safe and critical for improving posttreatment outcomes among adult patients with cancer after BMT [17], but far fewer studies exist for children and adolescent patients [18-20]. Starting an early exercise regime in the hospital is likely beneficial for optimizing health at admission to prepare the body for BMT, as well as for preventing physical deconditioning that can occur while admitted due to an immunocompromised status requiring isolation. However, there is a paucity of physical activity research among pediatric patients with BMT [21-24].

### Adulthood and Health Conditions

The transition from adolescence to adulthood is crucial for adopting healthy lifestyle behaviors, including physical activity, to manage and prevent health conditions. Pediatric cancer survivors have a high rate of severe, disabling, or life-threatening health conditions that can occur years after treatment [25]. In contrast, increased cardiorespiratory fitness and physical activity levels among pediatric cancer survivors have been linked with positive outcomes, such as a lower risk of obesity and cardiometabolic complications, improved overall health-related quality of life and social function, less cancer worry, and improved mental health (eg, cognitive function, depression, and self-body image) [26,27]. Another key impetus for promoting physical activity is to treat, manage, and prevent accelerated frailty that occurs among pediatric cancer survivors. The prevalence of frailty among this group has been found to be like that among adults 65 years and older [28].

### Limited Physical Activity Options and Gaps in Knowledge

Research findings addressing physical activity interventions are nonconfirmatory and are not generalizable to the general pediatric cancer population. A recent scoping review identified only 8 RCTs of physical activity for pediatric cancer survivors [29]. A systematic review identified only 2 studies that were of high enough quality to be reviewed, indicating a need for large RCTs [30]. Hospital physical activity programs have been promising but sparse [21-24]. The limitations of these studies included small sample sizes, underpowered study designs, in-person supervision by a specialist that is difficult to access in real-world settings, and limited accessible modalities for individuals with restricted mobility and functional capacity. Moreover, the modalities of aerobic exercise training were primarily walking, jogging, and bicycling, which are difficult to perform for patients with cancer who are in the early stages of treatment recovery, are in concomitant infection isolation, are severely deconditioned, or have mobility impairments. There is a dire need for early hospital physical activity modalities that are low burden on hospital staff, which can be delivered across multiple sites to improve health outcomes among pediatric patients with cancer who undergo BMT.

### Special Needs for Physical Activity

Physical performance limitations are prevalent among pediatric cancer survivors [6]. Relevant to physical activity performance, cancer survivors have poor ambulatory function [31,32] and energy expenditure [6], and have 24% greater odds of a mobility disability [31], all of which make prolonged walking and cycling difficult to perform for prolonged periods. Given that improvements to cardiometabolic health require gradual physiological adaptations following 1-3 months of exercise training [33-35], it is important to identify programs that are not only enjoyable and accessible but also effective in maintaining the interest of pediatric cancer survivors while addressing the varied needs within this group. There is an even greater need for those who undergo surgery, radiation therapy, or chemotherapy who have experienced damage to body structures or system functions and have the greatest risk for physical disability [6]. To address this need, two lessons can be learned from the field of adapted physical activity, which represents physical activity and exercise participation among people with disabilities. First, the field includes over 30 years of research in modifying and developing exercise movements and techniques to accommodate a wide variety of functional needs [36]. Second, RCTs have transitioned from a focus on multisite, supervised, in-person interventions to remote telehealth delivery [36], accommodating the low density of people who live near disability exercise specialists. Before this change, average sample sizes were 30 people per trial [36]; this is similar to current physical activity trials in pediatric cancer rehabilitation [29].

### Active Video Gaming is an Accessible and Serious Exercise

A promising area of study is the incorporation of active video gaming technology with telehealth procedures. A key benefit of active video gaming is that it provides a high level of enjoyment for children and youth. Active video gaming using an extended reality (ER) head-mounted display, the Meta Quest 3 (Reality Labs), has emerged as a promising active video gaming alternative due to its affordability and high-quality visual experiences in both virtual reality (the person is immersed in a digital environment) and mixed reality (digital objects and environments are projected into the real world through pass-through cameras). Studies have demonstrated that active video gaming with the Quest 2 (a fully virtual reality device) can achieve moderate exercise levels and provide a safe activity for various user groups, including child wheelchair users [37] and patients in intensive care [38]. In addition, virtual reality programs with psychological intervention have been found to be easy to use in clinics [39] and can improve psychological well-being among patients with cancer [40,41], which is critical given the psychological distress that can occur during and after cancer treatment [42,43]. However, to the best of our knowledge, there is no program that includes both mental health and physical activity in an intervention designed for deconditioned pediatric patients with cancer, particularly while supporting the transition across admission, BMT, and home.

### Telehealth Coaching Enhances Adherence

Home-based telehealth programs that incorporate “virtual” behavioral coaching (telecoaching) are a desirable approach for promoting nonsupervised exercise behavior within the community. Telehealth interventions that use remote communication technology bypass barriers related to lack of access, time, and nearby specialists, which are common for both children with disabilities [44] and those who have had cancer [45,46]. According to the Supportive Accountability Theory [47], programs that use telehealth technology can foster strong intervention adherence by providing a sense of accountability through remote monitoring and strong relationships with specialized health professionals. The addition of behavioral coaching strategies such as goal setting, confidence building, setting reasonable expectations, and understanding benefits, underpinned by theories such as the Social Cognitive Theory [48], have been found to enhance the likelihood of promoting exercise behavior among people with disabilities [49] and cancer survivors [50]. Social Cognitive Theory provides a targeted approach toward promoting exercise behavior through 4 constructs: self-efficacy (perceived control over one’s behaviors), outcome expectations, sociostructural factors (facilitators and barriers), and goals [48,51]. A physical activity intervention that uses telehealth technology, active video gaming in the ER, and a minimal dose of behavioral coaching has the potential to promote sustainable exercise behavior among large groups of pediatric cancer survivors.

This study tests a nearly full telehealth protocol with an ER program that includes physical activity and mental health intervention using “off-the-shelf” supplies. The study will inform a larger RCT through the following aims: (1) to examine the preliminary effects of an 8-week ER gaming telehealth program on physical activity participation among 16 pediatric patients with cancer after transplantation; (2) to explore potential program effects on key physical and mental health outcomes; and (3) to evaluate the feasibility of the program and its telehealth delivery through acceptability and safety.

## Methods

### Study Design and Overview

**Figure 1:**
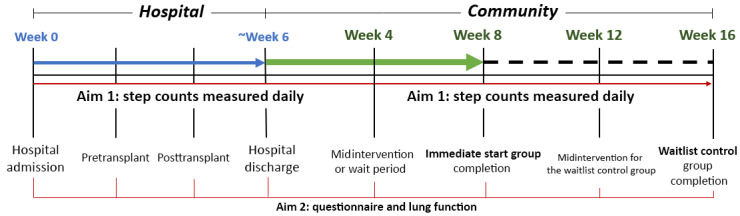
Study procedures for both the immediate start and waitlist control groups.

This phase I pilot feasibility RCT will include a 2-arm, parallel-group design to examine the preliminary effects of an ER telegaming intervention on physical activity levels among pediatric patients with cancer from a children’s hospital, compared with a waitlist control (WC). The design also includes a single-group crossover, where the WC group will receive the ER intervention after their designated wait period. The design is displayed in Figure 1. The project will include 16 pediatric patients with cancer from a single hospital who will complete the study (enrollment targets are 8 in year 1 and 8 in year 2).

This study has 2 groups, an immediate start (IS) group and a WC group. The IS group will start the intervention as soon as possible (ideally, pretransplantation within admission for BMT). Once the IS group is discharged from the hospital, they will continue the 8-week intervention at home with remote telehealth support. The anticipated hospital stay will last approximately 6 weeks. Thus, the total duration of time for the IS group will be 14 weeks (6 weeks in the hospital and 8 weeks of home intervention). The WC group will undergo the usual care within the hospital (approximately 6 weeks) and for 8 weeks after they are discharged. A total of 8 weeks after hospital discharge, WC participants will start the same 8-week intervention provided to the IS group. Data will be collected remotely throughout the study through digital monitoring technology and remote testing procedures. The total duration for WC participants in the study will be 22 weeks.

### Participants

The inclusion and exclusion criteria are listed in Textbox 1.

**Inclusion and exclusion criteria.**

**Inclusion criteria**

- Patient at the children’s hospital where the study is being conducted
- ≥8 years of age
- Medical diagnosis of malignancy or nonmalignant condition requiring hematopoietic stem cell transplant
- A caregiver who can assist the child with the study surveys and forms
- Access to a Wi-Fi Internet connection in the home through mobile phone or tablet computer

**Exclusion criteria**

- Complete blindness or deafness
- Inability to operate the handheld controllers
- Susceptibility to motion sickness in extended reality
- Inability to communicate in English

### Screening and Recruitment

Pediatric patients with cancer are recruited from cancer clinics within the pediatric hematology-oncology department of the hospital. The recruitment strategy is physician referral through word of mouth and study flyers. Specifically, a pediatric cancer rehabilitation medicine physician team member will attend weekly BMT team meetings to scout for potentially suitable patients for the study. Prospective participants will be referred in the team meeting to the rehabilitation medicine physician, who will inform the research staff to approach the patient in their room for screening and recruitment. Research staff will screen prospective participants for the eligibility criteria and obtain informed consent and assent for participation.

### Randomization and Clinical Trial Considerations

Participants will be randomized into 1 of 2 groups—IS or WC (8 per group)—with a 1:1 allocation ratio using a permuted block randomization approach. Participants will be evenly stratified into the IS or WC group based on sex. The randomization sequence will be generated by the project statistician using a computer-generated random schedule in a permuted block (SAS, SAS Institute Inc., version 9.4). Data will be entered into a secure electronic database (REDCap [Research Electronic Data Capture], Vanderbilt University) by a research assistant. The project manager will unfold the randomization sequence for successfully enrolled participants into 1 of the 2 groups. Study outcomes will be assessed by a research assistant who will be blinded to group allocation (single-blinded trial design).

### Supplies

This study will include the following supplies:

1. Meta Quest 3 ER head-mounted display with handheld controllers, preinstalled with several active video games that incorporate age-appropriate themes and music and mindfulness gaming. The Quest allows gameplay in both virtual and mixed reality. The Quest will come equipped with accessories to enhance accessibility and usability for children with disabilities, namely, an aftermarket head strap to reduce frontal pressure on the face and pressure on the neck, and hand straps to allow gameplay for children with minimal grip strength.
2. Fitbit Flex 2 (Google) activity monitor and sleep tracker, which will be used to track daily steps.

### Intervention Procedures

In summary, key features of the intervention include (1) an ER exercise intervention that has been adapted for a variety of functional abilities and fitness levels; (2) theory-based behavioral telecoaching; and (3) brief mindfulness ER gaming.

### ER Intervention

IS participants will undergo an ER intervention that will last approximately 14 weeks in total. IS participants will start the ER intervention in the hospital, which will last approximately 6 weeks. They will then participate in an 8-week, home-based program with behavioral coaching. IS participants will be provided with a Quest headset within their hospital room to begin the intervention. In the hospital, IS participants will be prescribed to complete at least 15 minutes of exercise and 10 minutes of mindfulness gaming per day. Video games will be preinstalled on the headsets. Exercise games will include boxing, fitness games, and rhythmic movement-to-music games (eg, Beat Saber [Beat Games], Les Mills Body Combat [Odders], Synth Riders [Kluge Interactive], Creed: Rise to Glory [Survios]). Mindfulness gaming will include TRIPP (TRIPP, Inc.), which is a comprehensive, high-quality mindfulness ER experience that can be purchased in the digital Quest store. TRIPP includes an extensive archive of content with techniques related to breathing, meditation, yoga, pain distraction, and positive self-image. After hospital discharge, IS participants will be provided with a Quest headset and be prescribed to complete the same exercise prescription 3 times per week. The prescription will be gradually progressed through weekly behavioral coaching calls, with a goal of reaching 150 minutes of moderate-intensity ER exercise per week by the fifth week of the home intervention. The desired 150 minutes was chosen because it will likely exceed an exercise dose that could improve health in people with mobility disabilities who are physically deconditioned [52].

WC participants will receive the same intervention as those in the IS group. Participants will be given a Meta Quest 3 ER headset that will come preinstalled with the intervention games. However, WC participants will not receive the intervention until 8 weeks after hospital discharge. Thus, WC participants will be in the study for a total of approximately 22 weeks. The WC control group will undergo usual care for a total of 14 weeks (6-week hospital stay and 8-week wait period at home) before starting the 8-week intervention. Usual care consists of inpatient and outpatient rehabilitation.

### Home-Based Behavioral Coaching

The home-based intervention will include behavioral coaching through phone calls with both the child and caregiver. The behavioral coaching calls will aim to enhance adherence by providing a sense of accountability [47], basic exercise knowledge, gameplay tips to increase mastery, and gradually promoting walking or community activity as a supplement to video gameplay. The rationale for promoting community physical activity is to transform a patient into a participant in their community by ER gameplay in the early intervention period to improve fitness levels that support gradual reintegration into the community. Calls will last approximately 15 minutes and will be provided weekly. Caregivers will be included in the calls since caregiver knowledge and attitude are determinants of participation.

The coaching calls will include behavior change strategies framed within the Social Cognitive Theory [48], including goal setting, confidence building, setting reasonable expectations, and understanding benefits. These strategies have been found to enhance the likelihood of promoting exercise behavior among people with disabilities [49] and cancer survivors [50]. Social Cognitive Theory provides a targeted approach toward promoting exercise behavior through 4 constructs: self-efficacy (perceived control over one’s behaviors), outcome expectations, sociostructural factors (facilitators and barriers), and goals [48,51]. The coaching calls will be delivered by the lead interventionists (AW and BL).

### Measures

All measures for this study will attempt to be conducted remotely, when possible. There are a total of 5 data collection time points that will be compared between groups, which are baseline (week 0), posttransplant (~week 3), hospital discharge (~week 6), the middle of the home intervention or wait (week 10), and postintervention (after week 14). WC participants will undergo 2 additional measurements, a mid- (week 18) and postintervention measure (week 22) of their home intervention. The first data collection visit will occur in person, and participants will be guided through the measurement instruments. The remaining 4 data collections will be conducted through phone or videoconference. The rationale for doing so is to simulate a remotely conducted trial and thus allow for easier replication in a future multisite trial. In addition, research staff would not have to track down participants in various clinics throughout their treatment and outpatient rehabilitation phases.

#### Aim 1 (Primary Outcome): Physical Activity

Physical activity level will be measured by steps per day using a Fitbit Flex 2 wrist-worn tri-axial accelerometer. The participants from both the IS and WC groups will be equipped with the Fitbit Flex as soon as they join the study. IS participants will wear the Fitbit Flex and daily steps will be measured until they complete the entire study (hospital phase, weeks 0-6; home intervention phase, weeks 7-14). WC participants will wear the Fitbit Flex, and daily steps will be measured until they complete all 3 phases that are assigned to their group: hospital phase, weeks 0-6; home wait period, weeks 7-14; and home intervention, weeks 15-22. The Fitbit Flex has evidence to support good-to-excellent validity and reliability for measuring step counts across a variety of populations, both general [53] and clinical [54], and moderate data to support validity for pediatric patients with cancer [55]. Additional rationales for the Fitbit Flex include ease of use, robust memory (30 days), water resistance, the small size of the monitor unit, and low cost. Although Actigraph GT3x monitors have more evidence to support their use among pediatric patients with cancer and are considered the gold standard [55], we chose not to use them due to their short storage capacity (42 days max) and an inability for both the participant and the telecoach to view and monitor their steps (app and web-based feedback; a critical component of coaching). Step counts are inversely associated with mortality [56], as well as fatigue and cumulative symptoms among patients with pediatric cancer [57].

#### Aim 2: Health Outcomes

Health outcomes will be measured upon joining the study at baseline (week 0), posttransplant (~week 3), before discharge (~week 6), midintervention or wait period (~week 10), and at the end of the 8-week home intervention or wait period (~week 14). WC participants will undergo additional health measurements at week 18 (midintervention) and after week 22 (postintervention). Health outcomes will include self-reported global health and lung function.

Self-reported global health will be measured by the National Institutes of Health (NIH) Patient-Reported Outcomes Measurement Information System (PROMIS) Pediatric Global Health Scale Short Form 7+2 (PGH-7+2) survey [58,59]. The original PGH-7 has been tested for validity among representative samples in a variety of pediatric clinical groups [59-61]. The PGH-7+2 includes 7 questions from the original scale that ask about general health, general quality of life, general physical health, general mental health, the frequency of feeling sad, the frequency of having fun with friends, and the frequency of caregivers listening to ideas, along with 2 additional questions: the frequency of feeling and trouble sleeping. Lower scores indicate a poorer response. The PGH-7+2 is scored by summing the values of the first 7 questions that correspond to global health, which will be transformed into a raw T-score and an SE using tables provided by survey developers. The last 2 questions are not scored as part of global health; instead, they are reported as raw response scores. A T-score of 50 is the average for the US population, with an SD of 10. Research staff will have 2 versions of the PGH-7+2 available to account for differing abilities and preferences: a caregiver proxy report version and a pediatric self-report version. Lung function will be measured by a test of forced expiratory volume using a peak flow meter. The participants will be instructed to forcefully exhale as hard as they can into the peak flow meter. The test will consist of 3 trials, where the average will represent the final value for lung function.

#### Aim 3: Intervention Acceptability

Intervention acceptability will be measured through participant engagement. Participant engagement outcomes will include enrollment, dropout, adverse events, and attendance rates. Attendance to the ER prescription will be expressed as a percentage of the minutes of gaming performed, divided by the minutes prescribed. Minutes of exercise will be recorded automatically by the headset in the built-in Meta Quest Move app. Meta Quest Move tracks active minutes of play each day, which can be archived for a year and be accessed either in the headset or through the Meta mobile phone app. The Move app also allows users to set weekly goals that include built-in notification features on goal progress. Research staff will set up Move goals to match the dose of intervention prescribed in the hospital and instruct caregivers to modify the goals when participants exercise at home.

### Statistical Analysis

Descriptive statistics, including means, SDs, and CIs, will be obtained for all study variables, and longitudinal smoothed scatterplots will be examined to identify temporal trends between groups, including the presence of potentially important nonlinear time trends. For aim 1 and aim 2 (measures of outcomes), outcomes will be modeled using all available measures, using mixed models with each participant included as a random effect to account for the repeated measures. Changes in means will be estimated with confidence intervals and compared between groups at 5 time points, which are baseline (week 0), posttransplant, hospital discharge (~week 6), midintervention or wait period (week 10), and postintervention (week 14) using estimated marginal means (also called least squares means). For aim 3, estimates with 95% CIs will be obtained for enrollment, dropout, and adverse event rates. Statistical tests will be 2-sided and will be performed using a significance level of *P*<.05. Statistical analyses will be performed using R (R Development Core Team) version 4.3.1 or higher (R Foundation for Statistical Computing).

### Ethical Considerations

Before the enrollment of participants, this study was approved by the institutional review board of the University of Alabama at Birmingham. Written informed consent and assent documentation will be obtained from all participants before their engagement in the study. Participants will receive an electronic debit card that will be loaded for US $50 for each of the data collections completed. The IS group has 5 data collections, totaling US $250. The waitlist group has 7 data collections, totaling US $350. The rationale for this value is to account for the commitment and time required of both the child and caregiver to complete the study.

The protocol and informed consent and assent forms were approved by the Institutional Review Board for Human Use of the University of Alabama at Birmingham (IRB-300012601) on June 27, 2024. Prospective participants will provide written informed consent (signed by caregivers or patients aged 18 years or older) or assent documentation (signed by children under the age of 18 years) before joining the study. Consent and assent forms will be completed in-person during the baseline data collection visit.

## Results

The study was funded in February 2024. This study started on June 27, 2024. Recruitment procedures started on June 27, 2024. This protocol paper was submitted for review before the enrollment of the first study participant. All data are anticipated to be collected by February 2026. Full trial results are anticipated to be analyzed and submitted for publication by March 2026. The study’s anticipated end date is March 31, 2026.

## Discussion

### Overview

This study will investigate the preliminary efficacy of a clinic-to-community ER program for improving both physical and mental health among pediatric patients undergoing cancer rehabilitation. The program incorporates off-the-shelf supplies, and most study procedures are done through telecommunications, which are 2 elements that will enhance the likelihood of scaling up the program into a multisite clinical trial. A program that can remotely link patients with pediatric cancer with ER health specialists through the internet could benefit this population. Incidence rates of cancer among pediatric patients and young adults are far lower than incidence rates among middle-aged and older adults [62]. Moreover, pediatric patients with cancer have limited options to improve their physical activity and mental health levels, particularly during their stay in the hospital [29].

### Strengths and Limitations

Considering that this program can be remotely delivered, it has the potential to be carried forward in a scale-up RCT. This is important, considering that the average sample size for an RCT for pediatric patients with cancer has been found to be 24 people (7 studies, mean sample size=24, SD 9) [29]. This indicates a need for large, multisite trials. Another strength of this study is that the chosen games incorporate child-appropriate themes and music. Music-based interventions can improve mental health among pediatric patients with cancer, as demonstrated by improvements in anxiety, pain, depression, state of mind, self-esteem, and quality of life [63].

This is a preliminary pilot study with a high focus on participant engagement and a small sample size. Should this trial be successful in improving physical activity levels and health, future research efforts will be needed to confirm the study findings in larger sample sizes using objective biomarkers of health.

### Conclusions

Should the findings of this study suggest that the program can improve physical activity levels, global health, and lung function, the study may discover an innovative and scalable method of intervention among pediatric patients with cancer undergoing rehabilitation.

## Appendix

Multimedia Appendix 1 Peer-review report by Kaul Pediatric Research Institute - KPRI Grant Program - The University of Alabama at Birmingham (USA).

## Abbreviations

BMT: bone marrow transplant
ER: extended reality
IS: immediate start
NIH: National Institutes of Health
PGH-7+2: NIH PROMIS Pediatric Global Health scale Short Form 7+2
PROMIS: Patient-Reported Outcomes Measurement Information System
RCT: randomized controlled trial
REDCap: Research Electronic Data Capture
WC: waitlist control

## Data Availability

The datasets generated during and/or analyzed during this study will be made publicly available upon completion of the study and can be obtained from the corresponding author on reasonable request.

## References

[1] Steliarova-Foucher E, Colombet M, Ries LAG, Moreno F, Dolya A, Bray F, Hesseling P, Shin HY, Stiller CA (2017). International incidence of childhood cancer, 2001-10: a population-based registry study. Lancet Oncol.

[2] Gordon-Dseagu V, Devesa SS, Goggins M, Stolzenberg-Solomon R (2018). Pancreatic cancer incidence trends: evidence from the surveillance, epidemiology and end results (SEER) population-based data. Int J Epidemiol.

[3] Butler E, Ludwig K, Pacenta HL, Klesse LJ, Watt TC, Laetsch TW (2021). Recent progress in the treatment of cancer in children. CA Cancer J Clin.

[4] Ward E, DeSantis C, Robbins A, Kohler B, Jemal A (2014). Childhood and adolescent cancer statistics, 2014. CA Cancer J Clin.

[5] Tonorezos ES, Cohn RJ, Glaser AW, Lewin J, Poon E, Wakefield CE, Oeffinger KC (2022). Long-term care for people treated for cancer during childhood and adolescence. Lancet.

[6] Ness KK, Hudson MM, Ginsberg JP, Nagarajan R, Kaste SC, Marina N, Whitton J, Robison LL, Gurney JG (2009). Physical performance limitations in the Childhood Cancer Survivor Study cohort. J Clin Oncol.

[7] Segal R, Zwaal C, Green E, Tomasone JR, Loblaw A, Petrella T, Exercise G (2017). Exercise for people with cancer: a clinical practice guideline. Curr Oncol.

[8] Posadzki P, Pieper D, Bajpai R, Makaruk H, Könsgen N, Neuhaus AL, Semwal M (2020). Exercise/physical activity and health outcomes: an overview of Cochrane systematic reviews. BMC Public Health.

[9] Momma H, Kawakami R, Honda T, Sawada SS (2022). Muscle-strengthening activities are associated with lower risk and mortality in major non-communicable diseases: a systematic review and meta-analysis of cohort studies. Br J Sports Med.

[10] Antwi GO, Jayawardene W, Lohrmann DK, Mueller EL (2019). Physical activity and fitness among pediatric cancer survivors: a meta-analysis of observational studies. Support Care Cancer.

[11] Taskinen M, Saarinen-Pihkala UM, Hovi L, Lipsanen-Nyman M (2000). Impaired glucose tolerance and dyslipidaemia as late effects after bone-marrow transplantation in childhood. Lancet.

[12] Armenian SH, Sun CL, Kawashima T, Arora M, Leisenring W, Sklar CA, Baker KS, Francisco L, Teh JB, Mills G, Wong FL, Rosenthal J, Diller LR, Hudson MM, Oeffinger KC, Forman SJ, Robison LL, Bhatia S (2011). Long-term health-related outcomes in survivors of childhood cancer treated with HSCT versus conventional therapy: a report from the bone marrow transplant survivor study (BMTSS) and childhood cancer survivor study (CCSS). Blood.

[13] Lin YF, Lairson DR, Chan W, Du XL, Leung KS, Kennedy-Nasser AA, Martinez CA, Gottschalk SM, Bollard CM, Heslop HE, Brenner MK, Krance RA (2010). The costs and cost-effectiveness of allogeneic peripheral blood stem cell transplantation versus bone marrow transplantation in pediatric patients with acute leukemia. Biol Blood Marrow Transplant.

[14] Shimosato Y, Tanoshima R, Tsujimoto SI, Takeuchi M, Shiba N, Kobayashi T, Ito S (2020). Allogeneic bone marrow transplantation versus peripheral blood stem cell transplantation for hematologic malignancies in children: a systematic review and Meta-Analysis. Biol Blood Marrow Transplant.

[15] Carlson K, Smedmyr B, Bäcklund L, Simonsson B (1994). Subclinical disturbances in cardiac function at rest and in gas exchange during exercise are common findings after autologous bone marrow transplantation. Bone Marrow Transplant.

[16] Soubani AO, Miller KB, Hassoun PM (1996). Pulmonary complications of bone marrow transplantation. Chest.

[17] Morales-Rodriguez E, Pérez-Bilbao T, San Juan AF, Calvo JL (2022). Effects of exercise programs on physical factors and safety in adult patients with cancer and haematopoietic stem cell transplantation: a systematic review. Int J Environ Res Public Health.

[18] Munsie C, Ebert J, Joske D, Ackland T (2019). The benefit of physical activity in adolescent and young adult cancer patients during and after treatment: a systematic review. J Adolesc Young Adult Oncol.

[19] West SL, Gassas A, Schechter T, Egeler RM, Nathan PC, Wells GD (2014). Exercise intolerance and the impact of physical activity in children treated with hematopoietic stem cell transplantation. Pediatr Exerc Sci.

[20] Cheung AT, Li WHC, Ho LLK, Ho KY, Chan GCF, Chung JOK (2021). Physical activity for pediatric cancer survivors: a systematic review of randomized controlled trials. J Cancer Surviv.

[21] San Juan AF, Fleck SJ, Chamorro-Viña C, Maté-Muñoz JL, Moral S, García-Castro J, Ramírez M, Madero L, Lucia A (2007). Early-phase adaptations to intrahospital training in strength and functional mobility of children with leukemia. J Strength Cond Res.

[22] San Juan AF, Fleck SJ, Chamorro-Viña C, Maté-Muñoz JL, Moral S, Pérez M, Cardona C, Del Valle MF, Hernández M, Ramírez M, Madero L, Lucia A (2007). Effects of an intrahospital exercise program intervention for children with leukemia. Med Sci Sports Exerc.

[23] San Juan AF, Chamorro-Viña C, Moral S, Fernández del Valle M, Madero L, Ramírez M, Pérez M, Lucia A (2008). Benefits of intrahospital exercise training after pediatric bone marrow transplantation. Int J Sports Med.

[24] Smith C, Farhat R, Fern-Buneo A, Purrington H, Cobb E, Matson L, Kang P, Beebe K, Campbell C, Schwalbach C, Salzberg D, Miller H, Adams R, Ngwube A (2022). Effects of an exercise program during pediatric stem cell transplantation: a randomized controlled trial. Pediatr Blood Cancer.

[25] Oeffinger KC, Mertens AC, Sklar CA, Kawashima T, Hudson MM, Meadows AT, Friedman DL, Marina N, Hobbie W, Kadan-Lottick NS, Schwartz CL, Leisenring W, Robison LL (2006). Chronic health conditions in adult survivors of childhood cancer. N Engl J Med.

[26] Lemay V, Caru M, Samoilenko M, Drouin S, Alos N, Lefebvre G, Levy E, Lippé S, Marcil V, Sultan S, Bertout L, Krajinovic M, Laverdière C, Raboisson MJ, Sinnett D, Andelfinger G, Curnier D (2019). Prevention of Long-term adverse health outcomes with cardiorespiratory fitness and physical activity in childhood acute lymphoblastic leukemia survivors. J Pediatr Hematol Oncol.

[27] Paxton RJ, Jones LW, Rosoff PM, Bonner M, Ater JL, Demark-Wahnefried W (2010). Associations between leisure-time physical activity and health-related quality of life among adolescent and adult survivors of childhood cancers. Psychooncology.

[28] Hayek Samah, Gibson Todd M, Leisenring Wendy M, Guida Jennifer L, Gramatges Maria Monica, Lupo Philip J, Howell Rebecca M, Oeffinger Kevin C, Bhatia Smita, Edelstein Kim, Hudson Melissa M, Robison Leslie L, Nathan Paul C, Yasui Yutaka, Krull Kevin R, Armstrong Gregory T, Ness Kirsten K (2020). Prevalence and Predictors of Frailty in Childhood Cancer Survivors and Siblings: A Report From the Childhood Cancer Survivor Study. J Clin Oncol.

[29] Caru M, Levesque A, Rao P, Dandekar S, Terry C, Brown V, McGregor L, Schmitz K (2022). A scoping review to map the evidence of physical activity interventions in post-treatment adolescent and young adult cancer survivors. Crit Rev Oncol Hematol.

[30] Crowder SL, Buro AW, Stern M (2022). Physical activity interventions in pediatric, adolescent, and young adult cancer survivors: a systematic review. Support Care Cancer.

[31] Salerno EA, Saint-Maurice PF, Willis EA, Moore SC, DiPietro L, Matthews CE (2021). Ambulatory function and mortality among cancer survivors in the NIH-AARP diet and health study. Cancer Epidemiol Biomarkers Prev.

[32] Wilson CL, Gawade PL, Ness KK (2015). Impairments that influence physical function among survivors of childhood cancer. Children (Basel).

[33] Lin X, Zhang X, Guo J, Roberts CK, McKenzie S, Wu WC, Liu S, Song Y (2015). Effects of exercise training on cardiorespiratory fitness and biomarkers of cardiometabolic health: a systematic review and meta-analysis of randomized controlled trials. J Am Heart Assoc.

[34] Ford ES (2002). Does exercise reduce inflammation? Physical activity and C-reactive protein among U.S. adults. Epidemiology.

[35] Snowling NJ, Hopkins WG (2006). Effects of different modes of exercise training on glucose control and risk factors for complications in type 2 diabetic patients: a meta-analysis. Diabetes Care.

[36] Lai B, Young HJ, Bickel CS, Motl RW, Rimmer JH (2017). Current trends in exercise intervention research, technology, and behavioral change strategies for people with disabilities: a scoping review. Am J Phys Med Rehabil.

[37] Lai B, Davis D, Narasaki-Jara M, Hopson B, Powell D, Gowey M, Rocque BG, Rimmer JH (2020). Feasibility of a commercially available virtual reality system to achieve exercise guidelines in youth with spina bifida: mixed methods case study. JMIR Serious Games.

[38] Lai B, Powell M, Clement AG, Davis D, Swanson-Kimani E, Hayes L (2021). Examining the feasibility of early mobilization with virtual reality gaming using head-mounted display and adaptive software with adolescents in the pediatric intensive care unit: case report. JMIR Rehabil Assist Technol.

[39] Tennant M, McGillivray J, Youssef GJ, McCarthy MC, Clark TJ (2020). Feasibility, acceptability, and clinical implementation of an immersive virtual reality intervention to address psychological well-being in children and adolescents with cancer. J Pediatr Oncol Nurs.

[40] Sansoni M, Malighetti C, Riva G (2022). Psychological and educational interventions among cancer patients: a systematic review to analyze the role of immersive virtual reality for improving patients? Well-being.

[41] Tennant M, Youssef GJ, McGillivray J, Clark TJ, McMillan L, McCarthy MC (2020). Exploring the use of immersive virtual reality to enhance psychological well-being in pediatric oncology: a pilot randomized controlled trial. Eur J Oncol Nurs.

[42] Nathan PC, Nachman A, Sutradhar R, Kurdyak P, Pole JD, Lau C, Gupta S (2018). Adverse mental health outcomes in a population-based cohort of survivors of childhood cancer. Cancer.

[43] Mertens AC, Gilleland Marchak J (2015). Mental health status of adolescent cancer survivors. COAYA.

[44] Martin Ginis KA, Ma JK, Latimer-Cheung AE, Rimmer JH (2016). A systematic review of review articles addressing factors related to physical activity participation among children and adults with physical disabilities. Health Psychol Rev.

[45] Mizrahi D, Wakefield CE, Simar D, Ha L, McBride J, Field P, Cohn RJ, Fardell JE (2020). Barriers and enablers to physical activity and aerobic fitness deficits among childhood cancer survivors. Pediatr Blood Cancer.

[46] Adamovich T, Watson R, Murdoch S, Giovino L, Kulkarni S, Luchak M, Smith-Turchyn J (2024). Barriers and facilitators to physical activity participation for child, adolescent, and young adult cancer survivors: a systematic review. J Cancer Surviv.

[47] Mohr DC, Cuijpers P, Lehman K (2011). Supportive accountability: a model for providing human support to enhance adherence to eHealth interventions. J Med Internet Res.

[48] Bandura A (2004). Health promotion by social cognitive means. Health Educ Behav.

[49] Ma JK, Martin Ginis KA (2018). A meta-analysis of physical activity interventions in people with physical disabilities: content, characteristics, and effects on behaviour. Psychology of Sport and Exercise.

[50] Stacey FG, James EL, Chapman K, Lubans DR (2016). Social cognitive theory mediators of physical activity in a lifestyle program for cancer survivors and carers: findings from the ENRICH randomized controlled trial. Int J Behav Nutr Phys Act.

[51] Motl RW, Pekmezi D, Wingo BC (2018). Promotion of physical activity and exercise in multiple sclerosis: importance of behavioral science and theory. Mult Scler J Exp Transl Clin.

[52] Martin Ginis KA, van der Ploeg HP, Foster C, Lai B, McBride CB, Ng K, Pratt M, Shirazipour CH, Smith B, Vásquez PM, Heath GW (2021). Participation of people living with disabilities in physical activity: a global perspective. Lancet.

[53] Jones D, Crossley K, Dascombe B, Hart HF, Kemp J (2018). Validity and reliability of the Fitbit Flex and Actigraph GT3X+ at jogging and running speeds. Int J Sports Phys Ther.

[54] Alharbi M, Bauman A, Neubeck L, Gallagher R (2016). Validation of Fitbit-Flex as a measure of free-living physical activity in a community-based phase III cardiac rehabilitation population. Eur J Prev Cardiol.

[55] Ha L, Mizrahi D, Wakefield CE, Cohn RJ, Simar D, Signorelli C (2021). The use of activity trackers in interventions for childhood cancer patients and survivors: a systematic review. J Adolesc Young Adult Oncol.

[56] Banach M, Lewek J, Surma S, Penson PE, Sahebkar A, Martin SS, Bajraktari G, Henein MY, Reiner Z, Bielecka-Dąbrowa A, Bytyçi I (2023). The association between daily step count and all-cause and cardiovascular mortality: a meta-analysis. Eur J Prev Cardiol.

[57] Withycombe JS, McFatrich M, Hinds PS, Bennett A, Lin L, Maurer SH, Lucas NR, Mann CM, Castellino SM, Baker JN, Reeve BB (2022). Can steps per day reflect symptoms in children and adolescents undergoing cancer treatment?. Cancer Nurs.

[58] Hays RD, Bjorner JB, Revicki DA, Spritzer KL, Cella D (2009). Development of physical and mental health summary scores from the patient-reported outcomes measurement information system (PROMIS) global items. Qual Life Res.

[59] Forrest CB, Bevans KB, Pratiwadi R, Moon J, Teneralli RE, Minton JM, Tucker CA (2014). Development of the PROMIS ® pediatric global health (PGH-7) measure. Qual Life Res.

[60] Luijten MAJ, Haverman L, van Litsenburg RRL, Roorda LD, Grootenhuis MA, Terwee CB (2022). Advances in measuring pediatric overall health: the PROMIS® pediatric global health scale (PGH-7). Eur J Pediatr.

[61] Forrest CB, Tucker CA, Ravens-Sieberer U, Pratiwadi R, Moon J, Teneralli RE, Becker B, Bevans KB (2016). Concurrent validity of the PROMIS® pediatric global health measure. Qual Life Res.

[62] Barr RD, Ries LAG, Lewis DR, Harlan LC, Keegan THM, Pollock BH, Bleyer WA (2016). Incidence and incidence trends of the most frequent cancers in adolescent and young adult Americans, including "nonmalignant/noninvasive" tumors. Cancer.

[63] González-Martín-Moreno M, Garrido-Ardila EM, Jiménez-Palomares M, Gonzalez-Medina G, Oliva-Ruiz P, Rodríguez-Mansilla J (2021). Music-based interventions in paediatric and adolescents oncology patients: a systematic review. Children (Basel).

