# Supplementary material for: Extended Reality Gaming for Exercise and Mindfulness Throughout Pediatric Cancer Rehabilitation: Protocol for a Randomized Controlled Trial
Source: JMIR Res Protoc. 2024 Dec 23;13:e64879. doi: 10.2196/64879 (PMC11704644; doi:10.2196/64879)
Supplement: Multimedia Appendix 1 [file resprot_v13i1e64879_app1.pdf]

KPRI Scientific Review

Please provide bullet points of the strengths and weaknesses. Score each section from 1-9 using the NIH review criteria (1 = exceptional, 9 = poor).

**REVIEWER:**	
**INVESTIGATOR:**	Byron Lai
**PROJECT TITLE:**	Virtual Reality Gaming to Improve Physical Activity and Pyschosocial Health Among Pediatric Cancer

OVERALL IMPACT/PRIORITY: | score | 2 |

Overall review of the scientific merit of the application. In particular consider the likelihood that the proposal will position the applicant for a competitive NIH or other major grant.

Strengths

- **Important topic with significant impact for a pediatric population, excellent team, tested methodology, strong likelihood of success and advancement to larger studies**

Weaknesses

- A few negligible points, including a question on the need for a control group vs. a larger subject group to accomplish study goals

SCORED REVIEW CRITERIA

SIGNIFICANCE: | score | 2 |

Will the proposed research yield insights into the fundamental processes that will improve child health?

Does the proposal contain pre-clinical (basic), quality improvement or clinical/translational research?

Strengths

- **Rehabilitation for pediatric cancer patients can make a life long difference**
- **The proposal makes a strong argument for why this method of delivery should be examined, and may be better than others that have been studied**

Weaknesses

- It is possible to argue that there is no good reason not to try this clinically, and this kind of rigorous study is not needed, as it seems obvious that physical activity with coaching will benefit these patients

INNOVATION: | score | 2 |

Does the application propose novel concepts, approaches or methodologies, aimed at improved child health?

Strengths

- **This is an unstudied method of encouraging physical activity in this population, built on evidence based methods done with other populations**

Weaknesses

- VR based exercise has become much more common since the pandemic. (however, no one has been doing this in a formal way in this population.)

APPROACH: | score | 2 |

Are the overall strategy, methodology, and analyses well-reasoned and appropriate to accomplish the specific aims of the project? Include a comment on whether the proposed work is appropriate for a 1-2 year project.

Strengths

- Methodology based on previously tested methods in different populations, including children with disabilities
- Measures have been tested for effectiveness
- 2 years is appropriate as recruitment will not be rapid, but can be achieved in that time frame

Weaknesses

- The need for a control group to accomplish the goals of the proposal is questionable. Subjects will do better than controls, that is obvious. The biggest question is how much they will improve. With the variance in the population, a larger sample size would be useful. This is easily achieved by running the program on the WC subjects, and then having 16 subjects. The investigators may be planning this but did not mention it.
- The proposal did not address the likely possibility of dropout due to illness. That may be considered part of the feasibility, but it would seem that there should be a definition of an early drop out point that would merit adding another subject.

INVESTIGATOR(S): **score | 1**

Is the Investigator well suited to complete the project? Include a comment on whether this is a junior or senior investigator.

Strengths

- **PI experienced with these studies, has appropriate clinical collaborators**

Weaknesses

- None

ENVIRONMENT: **score | 1**

Comment on the environment (including collaborators) and other available resources.

Strengths

- **The support systems in place for these kind of studies at this center are fantastic**

Weaknesses

- None

ADDITIONAL REVIEW CRITERIA

**BUDGET**
Appropriate
**Established Investigators**
For established investigators, is this an entirely new area of investigation, different from an applicant's current line of investigation?

Please use the following 1-9 scoring scale

Impact	Score	Descriptor	Additional Guidance on Strengths/Weaknesses
	1	Exceptional	Exceptionally strong with essentially no weaknesses

High	2	Outstanding	Extremely strong with negligible weaknesses
	3	Excellent	Very strong with only some minor weaknesses
Medium	4	Very Good	Strong but with numerous minor weaknesses
	5	Good	Strong but with at least one moderate weakness
	6	Satisfactory	Some strengths but also some moderate weaknesses
Low	7	Fair	Some strengths but with at least one major weakness
	8	Marginal	A few strengths and a few major weaknesses
	9	Poor	Very few strengths and numerous major weaknesses

Minor Weakness: An easily addressable weakness that does not substantially lessen impact
Moderate Weakness: A weakness that lessens impact
Major Weakness: A weakness that severely limits impact

KPRI Scientific Review

Please provide bullet points of the strengths and weaknesses. Score each section from 1-9 using the NIH review criteria (1 = exceptional, 9 = poor).

**REVIEWER:**	
**INVESTIGATOR:**	Byron Lai
**PROJECT TITLE:**	Virtual Reality Gaming to Improve Physical Activity and Psychological Health Am<br>Pediatric Cancer Rehabilitation Patients that Transition from Clinic to Community

OVERALL IMPACT/PRIORITY: score | 2

Overall review of the scientific merit of the application. In particular consider the likelihood that the proposal will position the applicant for a competitive NIH or other major grant.

Strengths

- Addresses great need for very vulnerable population.
- Strong evidence to support the methodology.
- Strong team with experience delivering interventions.
- Methods appear sound and appropriate for such this pilot RCT.
- Inclusion of behavioral coaching adds a great deal to this line of research.

Weaknesses

- Evidence exists already for other gaming devices

SCORED REVIEW CRITERIA

SIGNIFICANCE: score | 1

Will the proposed research yield insights into the fundamental processes that will improve child health? Does the proposal contain pre-clinical (basic), quality improvement or clinical/translational research?

Strengths

- Addresses great need for very vulnerable population.
- Uses safe yet effective approach.
- Inclusion of behavioral coaching adds a great deal to this line of research.

Weaknesses

- None

INNOVATION: score | 1

Does the application propose novel concepts, approaches or methodologies, aimed at improved child health?

Strengths

- Latest technology that is easy to use will be employed.
- Inclusion of behavioral coaching adds a great deal to this line of research.

Weaknesses

- None

APPROACH: score | 2

Are the overall strategy, methodology, and analyses well-reasoned and appropriate to accomplish the specific aims of the project? Include a comment on whether the proposed work is appropriate for a 1-2 year project.

Strengths

- Familiar and psychometrically sound outcome measures used.
- Inclusion of behavioral coaching adds a great deal to this line of research…behavioral intervention is theory based.

Weaknesses

- May be difficult to compare physical activity in hospital environment with community environment – but presence of a control group will help.

INVESTIGATOR(S): **score 1**

Is the Investigator well suited to complete the project? Include a comment on whether this is a junior or senior investigator.

Strengths

- Ample experience with this type of research

Weaknesses

- none

ENVIRONMENT: **score 1**

Comment on the environment (including collaborators) and other available resources.

Strengths

- Design is very adaptable to most any environment.
- Parents will be involved in design…to assure further adaptability.

Weaknesses

- none

ADDITIONAL REVIEW CRITERIA

**BUDGET**
Seems reasonable
**Established Investigators**
For established investigators, is this an entirely new area of investigation, different from an applicant's current line of investigation? This team (especially the PI) is experienced with the procedures.

Please use the following 1-9 scoring scale

Impact	Score	Descriptor	Additional Guidance on Strengths/Weaknesses
High	1	Exceptional	Exceptionally strong with essentially no weaknesses
	2	Outstanding	Extremely strong with negligible weaknesses
	3	Excellent	Very strong with only some minor weaknesses
	4	Very Good	Strong but with numerous minor weaknesses

Medium	5	Good	Strong but with at least one moderate weakness
	6	Satisfactory	Some strengths but also some moderate weaknesses
Low	7	Fair	Some strengths but with at least one major weakness
	8	Marginal	A few strengths and a few major weaknesses
	9	Poor	Very few strengths and numerous major weaknesses

Minor Weakness: An easily addressable weakness that does not substantially lessen impact
Moderate Weakness: A weakness that lessens impact
Major Weakness: A weakness that severely limits impact
